# THE ROLE OF AGE AND EXERCISE HISTORY IN AFFECTIVE RESPONSE TO ACUTE EXERCISE: PRELIMINARY RESULTS

**DOI:** 10.1093/geroni/igae098.3496

**Published:** 2024-12-31

**Authors:** Marta Stojanovic, Baylie Rushing, Madeleine Love, John Morris, Denise Head

**Affiliations:** Washington University in St. Louis, St. Louis, Missouri, United States; Washington University in St. Louis, St. Louis, Missouri, United States; Washington University in St. Louis, St. Louis, Missouri, United States; Washington University in St. Louis, St. Louis, Missouri, United States; Washington University in St. Louis, St. Louis, Missouri, United States

## Abstract

A more positive affective response to acute exercise has been linked to greater physical activity levels. Older adults might experience increased physiological symptoms (e.g., shallow breathing) during acute exercise and subsequently less positive affect post-exercise. Additionally, sedentary individuals were shown to have a more negative affective response. The goals of the study were to examine whether age and exercise history impact the affective response to exercise and its association with overall physical activity levels. Cognitively normal individuals from the Knight ADRC Adult Children Study (n=65; age range 44-85 years) completed an acute bout of moderate-intensity exercise. Affect was measured at baseline, immediately after, 15 minutes, and 30 minutes post-exercise. Participants also completed one week of actigraphy, which provided an objective estimate of their physical activity levels, and a self-report questionnaire about their exercise history over the past 10 years. While positive affect increased immediately post-exercise similarly across age (p=0.103), older individuals showed a quicker return to baseline (p=0.012). Age did not moderate the association between affect and physical activity (p>0.119). Exercise history did not predict affective response (p’s>0.847), however, individuals with greater exercise history showed a stronger association between a more positive affective response and greater physical activity levels (p< 0.001). These results suggest that older individuals might experience a less positive affective response to moderate-intensity exercise. Furthermore, exercise history might be a relevant moderator of the link between affective response and physical activity. Future studies should use a larger sample size and consider additional moderators, including cardiovascular health and sleep.

